# Functional Characterization of Serotonin *N*-Acetyltransferase in Archaeon *Thermoplasma volcanium*

**DOI:** 10.3390/antiox11030596

**Published:** 2022-03-21

**Authors:** Kyungjin Lee, Geun-Hee Choi, Kyoungwhan Back

**Affiliations:** 1 Department of Biotechnology, College of Agriculture and Life Sciences, Chonnam National University, Gwangju 61186, Korea; nicekj7@hanmail.net; 2 Nakdonggang National Institute of Biological Resources, 137 Donam 2-gil, Sangju-si 37242, Korea; ghchoi@nnibr.re.kr

**Keywords:** archaea, Ard1, *N*-acetylserotonin, *N*-acetyltyramine, synthetic genes, melatonin, rice seed size

## Abstract

Serotonin *N*-acetyltransferase is the penultimate enzyme in the melatonin biosynthetic pathway that catalyzes serotonin into *N*-acetylserotonin. Many *SNAT* genes have been cloned and characterized from organisms ranging from bacteria to plants and mammals. However, to date, no *SNAT* gene has been identified from Archaea. In this study, three archaeal *SNAT* candidate genes were synthesized and expressed in *Escherichia coli*, and SNAT enzyme activity was measured using their purified recombinant proteins. Two *SNAT* candidate genes, from Methanoregulaceae (Archaea) and *Pyrococcus furiosus*, showed no SNAT enzyme activity, whereas a *SNAT* candidate gene from *Thermoplasma volcanium* previously named *TvArd1* exhibited SNAT enzyme activity. The substrate affinity and the maximum reaction rate of TvSNAT toward serotonin were 621 μM and 416 pmol/min/mg protein, respectively. The highest amine substrate was tyramine, followed by tryptamine, serotonin, and 5-methoxytryptamine, which were similar to those of plant SNAT enzymes. Homologs of *TvSNAT* were found in many Archaea families. Ectopic overexpression of *TvSNAT* in rice resulted in increased melatonin content, antioxidant activity, and seed size in conjunction with the enhanced expression of seed size-related gene. This study is the first to report the discovery of *SNAT* gene in Archaea. Future research avenues include the cloning of *TvSNA*T orthologs in different phyla, and identification of their regulation and functions related to melatonin biosynthesis in living organisms.

## 1. Introduction

Melatonin is a universal molecule; it has been found in nearly all living organisms that have been tested for its presence [1,2]. Melatonin functions as a neurohormone, influencing circadian rhythms and seasonal behavioral changes, and acting as a potent antioxidant in animals. In plants, melatonin functions as an antioxidant as well as a signaling molecule, orchestrating various growth and defense processes, possibly via protein quality control and reactive oxygen species (ROS) scavenging mechanisms, although its role in circadian rhythm remains elusive [3,4,5,6,7]. In contrast to animals, which show a nocturnal peak in melatonin content, plants show nocturnal peaks in the content of melatonin metabolites, such as 2-hydroxymelatonin and cyclic 3-hydroxymelaotnin in rice plants [8,9]. Recently, 2-hydroxymelatonin was found to trigger ROS production in *Arabidopsis thaliana* leaves in a manner dependent on respiratory bursts of NADPH (nicotinamide adenine dinucleotide) oxidase [10], indicating the physiological significance of melatonin metabolites in plants. As in animals, these biological functions of melatonin are thought to be mediated by phytomelatonin receptors [11,12], whereas its antioxidant activity appears to be independent of phytomelatonin receptors [1,13].

The final two enzymes in the melatonin biosynthesis pathway are serotonin *N*-acetyltransferase (SNAT; also called arylalkyl *N*-acetyltransferase) and *N*-acetylserotonin *O*-methyltransferase [13,14]. SNAT is thought to be a rate-limiting enzyme in melatonin synthesis in both animals and plants [1]. In animals, *SNAT* evolved from Gram-positive bacteria [15], whereas in plants, *SNAT* evolved from cyanobacteria [2]. These independent evolutionary pathways have led a lack of significant homology among *SNAT* genes between animals and plants [16]. SNAT belongs to the GCN5-related *N*-acetyltransferase (GNAT) superfamily, members of which share a common acetyl-CoA binding domain and acetyl transfer mechanism; however, each family exhibits dramatically different substrate preferences [15,17]. In plants, it has been reported that the ectopic overexpression of *SNAT* either from animals or plants led to enhanced melatonin production followed by a series of physiological effects such as increased tolerance against biotic and abiotic stresses as well as yield increase [6,14].

Despite the functional characterization of a number of *SNAT* genes from bacteria [18], cyanobacteria [19], yeast [20], animals [21,22], and plants [23,24], *SNAT* from archaea have not been characterized to date. In common with other organisms, archaea are estimated to possess at least 200 GNAT proteins [25], of which two archaeal *GNAT* genes have been cloned and characterized according to crystallographic analysis of protein acetyltransferase activity [25,26]. However, SNAT enzyme activity has not been characterized in archaea to date. In this study, we employed two archaeal *GNAT* genes from *Pyrococcus furiosus* and *Thermoplasma volcanium* and one methanogenic archaea *GNAT* gene exhibiting similarity to rice *SNAT* [27] to investigate whether these archaeal GNAT proteins exhibit SNAT enzyme activity. This study is the first to report the identification of archaeal *SNAT* gene, providing a new avenue for investigating melatonin biosynthetic pathways in all kingdoms of living organisms.

## 2. Materials and Methods

### 2.1. Synthesis of Archaeal GNAT Genes

On the basis of protein information for three archaeal *GNAT* genes from *Pyrococcus furiosus* (GenBank accession no. NC_003413), *Thermoplasma volcanium* (NC_002689 or WP_010916271), and *Methanoregulaceae archaeon* PtaB.Bin152 (MVQF01000146), we manually designed corresponding nucleotide sequences with reference to the rice *SNAT2* codon [27]. Three synthetic *GNAT* genes were custom synthesized at Bioneer (Daejeon, South Korea).

### 2.2. Vector Construction and Purification of Recombinant Proteins

The full-length *GNAT* genes of *M. archaeon* (*MaGNAT*), *P. furiosus* (*PfGNAT*), and *T. volcanium* (*TvGNAT*) were initially amplified using polymerase chain reaction (PCR), and the primer sets are listed in Table S1. These were used with template plasmids containing each synthetic *GNAT* DNA (pBHA-MaGNAT, pBHA-PfGNAT, and pBHA-TvGNAT), which were provided by Bioneer. The initial *GNAT* PCR products were further amplified using a primer set containing the *attB* recombination sequence described in Table S1. These *GNAT* PCR products were cloned using Gateway recombination reactions into the pDONR221 vector (Invitrogen, Carlsbad, CA, USA), and then into the destination vector pET300/NT-DEST (Invitrogen) according to the manufacturer’s procedure. The three plasmids, pET300-MaGNAT, pET300-PfGNAT, and pET300-TvGNAT, were then transformed into *Escherichia coli* strain BL21(DE3) (Invitrogen). The seed culture (10 mL) was cultured overnight in the presence of the antibiotic ampicillin (50 mg/L) and inoculated into 100 mL of Terrific Broth medium, consisting of 20 g/L bacto-tryptone, 24 g/L bacto-yeast extract, 4 mL/L glycerol, and phosphate buffer (0.017 M KH_2_PO_4_ and 0.072 M K_2_HPO_4_). The culture was incubated at 37 °C until the optical density of the *E. coli* culture at 600 nm reached 1.0. After the addition of 1 mM isopropyl-β-d-1-thiogalactopyranoside (Sigma, St. Louis, MO, USA), the culture was grown at 28 °C and shaken at 180 rpm for 5 h. Purification procedures were performed using affinity (Ni^2+^) chromatography (Qiagen, Tokyo, Japan), according to the manufacturer’s recommendations.

### 2.3. Homology and Phylogenetic Analysis

Amino acid (aa) sequence homology analysis was performed using the BLASTp tool (National Library of Medicine, Bethesda, MD, USA), and non-redundant protein sequences databases of the National Center for Biotechnology Information (http://www.ncbi.nlm.nih.gov/, accessed on 24 April 2019). Phylogenetic analysis was performed using the BLAST-Explorer program (version 2, Information Genomique & Structurale, Marseille, France) [28].

### 2.4. SNAT Enzyme Kinetics Measurements

Each purified recombinant GNAT protein was incubated in a total volume of 100 µL containing 0.5 mM serotonin (or other substrates) and 0.5 mM acetyl-CoA in 100 mM potassium phosphate at pH 8.8 and 45 °C for 1 h; pH and temperature were varied in some samples. Reaction products such as *N*-acetylserotonin and melatonin were subjected to high-performance liquid chromatography (HPLC) as described previously [23]. Substrate affinity (*K*_m_) and the maximum reaction rate (*V*_max_) were calculated using Lineweaver–Burk plots. Protein concentrations were determined using the Bradford method and a protein assay dye (Bio-Rad, Hercules, CA, USA). These analyses were performed in triplicate.

### 2.5. Transgenic Rice Plants Overexpressing TvSNAT

To obtain transgenic rice plants overexpressing the synthetic *TvSNAT* gene, their full-length sequences were amplified by PCR using specific primers (Table S1) using the synthetic cDNA gene as a template. The initial PCR products were further amplified using a second primer set containing 14 nt of the *attB* sequence using the initial PCR product as a template. Secondary PCR products were gel purified and cloned into the pDONR221 gateway vector (Invitrogen) via BP recombination. The pDONR221-TvSNAT gene entry vector was then recombined with the pIPKb002 destination vector [29] via LR recombination to yield the pIPKb002-TvSNAT binary plasmid. Constitutive expression in rice transgenic lines was ensured by the maize ubiquitin promoter.

The binary vector plasmid was transformed into *Agrobacterium tumefaciens* LBA4404, followed by transformation into rice as described previously [30].

### 2.6. Quantification of Melatonin and Radical Scavenging Activity Using the DPPH Method

Melatonin levels in rice seedlings were quantified by HPLC using a fluorescence detection system (Waters, Milford, MA, USA) as described previously [9]. As for seed melatonin quantification, rough rice seeds were first imbibed for 24 h followed by chloroform extraction as performed in seedling samples. As for antioxidant activity measurement, rough rice seeds were employed. The radical scavenging activity was measured using the 1,1-diphenyl-2-picrylhydrazyl (DPPH) method as described previously [31]. In brief, rough rice seeds (0.2 g), which were imbibed for 24 h, were extracted with 1 mL methanol and spun down for 5 min at 12,000× *g*. The resulting supernatants of methanolic extracts (0.1 mL) were mixed with 0.9 mL of 0.15 mM DPPH solution dissolved in methanol at 27 °C for 20 min. The control was prepared as above without any extract. Radical scavenging activity was expressed as a percentage of inhibition and was calculated using the following numerical formula: % radical scavenging activity = (control optical density (OD) − sample OD/control OD) × 100.

### 2.7. Statistical Analyses

All data analyses were performed using the IBM SPSS Statistics 23 software (IBM Corp., Armonk, NY, USA). Data are reported as means ± standard deviation; significant differences were evaluated at *p* < 0.05 according to Tukey’s post hoc honest significant difference test.

## 3. Results

### 3.1. Codon-Optimized Synthesis of Three Archaeal GNAT Genes

Three archaeal *GNAT* genes were synthesized on the basis of aa sequence data from the GenBank database (http://www.ncbi.nlm.nih.gov/, accessed on 27 May 2019; Table S2).

**Figure 1:**
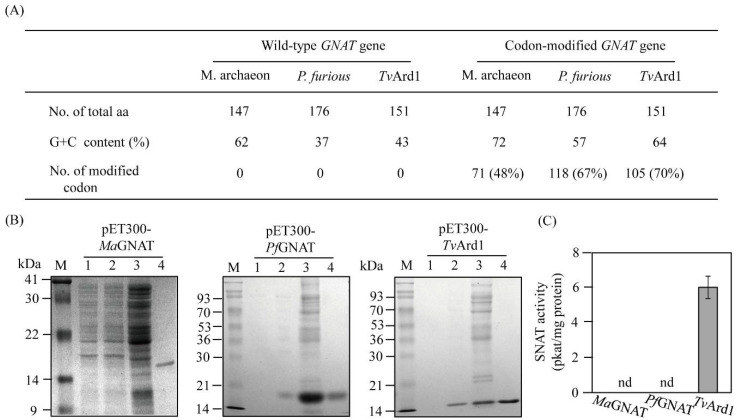
Summary of synthetic archaeal *GNAT* genes and recombinant GNAT protein purification. (**A**) Modification of archaeal *GNAT* genes. (**B**) Purification of N-terminal His × 6-tagged GNAT proteins. (**C**) SNAT activity measurements. Bacterial host strain BL21 (DE3) cells harboring the pET30-GNAT plasmids were incubated with isopropyl β-d-1-thiogalactopyranoside (IPTG) for 5 h at 28 °C. M, molecular mass standards; lane 1, total proteins in 15-µL aliquots of bacterial culture without IPTG; lane 2, total proteins in 15-µL aliquots of bacterial culture with IPTG; lane 3, 20 µg soluble protein; lane 4, 5 µg protein purified by affinity chromatography. Protein samples were separated using 12% sodium dodecyl sulfate–polyacrylamide gel electrophoresis and stained with Coomassie blue. SNAT enzyme activity was measured according to *N*-acetylserotonin production in the presence of 0.5 mM serotonin at 45 °C and pH 8.8.

Codon selection was performed on the basis of rice *SNAT2* codon preference for further overexpression in the rice genome. Because the rice *SNAT2* gene (GenBank accession no. AK068156) contains high G+C content (70%), the G+C contents of the synthetic genes of the three archaeal *GNAT* genes increased by 17% on average (Figure 1A). The *GNAT* gene from *M. archaeon* PtaB.Bin152 (*MaGNAT*) was selected due to its high aa sequence identity to rice SNAT2 (>45%) in the acetyl-CoA binding region, whereas there is little homology in the arylalkylamine binding region of GNAT family members [15]. Two other *GNAT* genes from *Pyrococcus furiosus* (*PvGNAT*) and *Thermoplasma volcanium* (*TvGNAT* or *TvArd1* (*arrest-defective-1*)) were chosen on the basis of their *N*-acetyltransferase (NAT) enzyme activity and successful expression in *Escherichia coli* [25,26]. The three codon-optimized archaeal *GNAT* genes were expressed as fusion proteins for the N-terminal hexa-histidine tag, followed by Ni^2+^ affinity purification (Figure 1B). All three GNAT proteins were successfully purified and subjected to SNAT enzyme assay analysis. MaGNAT and PfGNAT showed no SNAT enzyme activity, whereas TvGNAT exhibited high SNAT enzyme activity (6 pkat/mg protein) at 45 °C and pH 8.8 (Figure 1C). TvGNAT was previously annotated as an NAT Ard1 protein (TvArd1), which acetylates N-terminal residues of proteins such as *T. volcanium* Alba [26]. These data indicate that TvArd1 (now named TvSNAT) exhibits acetylation activity in transferring an acetyl group from acetyl-coenzyme A into both protein and serotonin. The optimum growth temperature of *T. volcanium* is approximately 60 °C, which is the lowest reported among Archaea [32]. 

### 3.2. Enzyme Kinetics of TvSNAT

**Figure 2:**
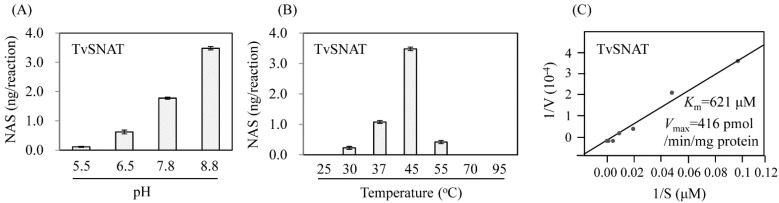
SNAT enzyme activity catalyzing the conversion of serotonin to *N*-acetylserotonin as a function of (**A**) pH and (**B**) temperature. (**C**) Determination of substrate affinity (*K*_m_) and maximum reaction rate (*V*_max_) values of TvSNAT (also called TvArd1). TvSNAT (1 µg) was incubated at a range of pH values and temperatures for 30 min. *K*_m_ and *V*_max_ values were determined using Lineweaver–Burk plots. In vitro enzymatic *N*-acetylserotonin products were measured by high-performance liquid chromatography. Data are the means ± standard deviation (*n* = 3).

To investigate the features of the TvSNAT enzyme (TvArd1), we performed experiments to determine their optimum pH and temperature. The highest SNAT activity was observed at pH 8.8 (Figure 2), similar to those of many plant SNAT proteins [23,27,33,34]. TvSNAT also showed optimum activity at temperatures ranging from 45 to 55 °C, similar to plant SNAT proteins [24]. By contrast, animal SNAT proteins have an optimum pH of 6.7 [21,22] and optimal temperature of approximately 37 °C [21,22]. The *K*_m_ and *V*_max_ values of TvSNAT were 621 μM and 416 pmol/min/mg protein, respectively. The *K*_m_ value of TvSNAT was similar to those of many other plant SNAT proteins [24], but different from those of SNAT proteins in animals such as human (1350 μM) and sheep (125 μM) [22]. On the basis of these data enzymatic features, we conclude that the TvSNAT protein is more closely related to plant SNAT proteins than to animal SNAT proteins. The high optimum temperature of TvSNAT enzyme activity was closer to the high optimum growth temperature of *T. volcanium* than to the low optimum temperature (25 °C) of protein NAT activity of TvArd1 [26], suggesting that TvArd1 shows SNAT activity in preference to NAT activity in vitro, although in vivo evidence is required. 

### 3.3. Substrate Specificity

**Figure 3:**
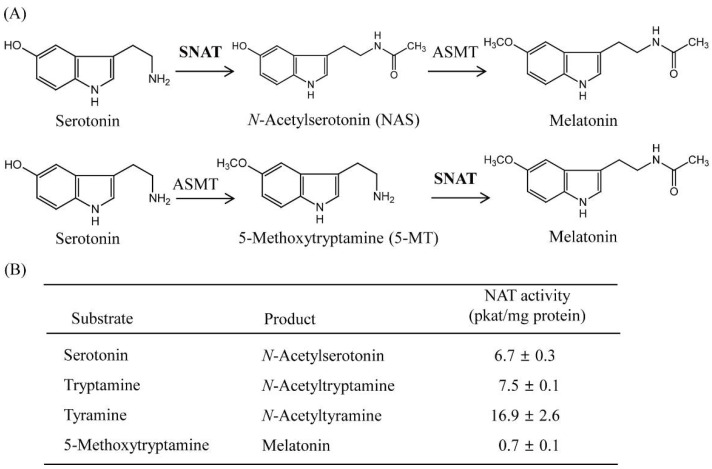
Schematic diagram of the SNAT reaction and substrate preference. (**A**) Enzymatic reaction of SNAT in two different melatonin biosynthetic pathways. (**B**) TvSNAT enzyme activity measurements for various substrates. SNAT enzyme activity was measured in the presence of 0.5 mM of each substrate at 45 °C and pH 8.8. Data are the means ± standard deviation (*n* = 3). ASMT, *N*-acetylserotonin *O*-methyltransferase; SNAT, serotonin *N*-acetyltransferase.

**Figure 4:**
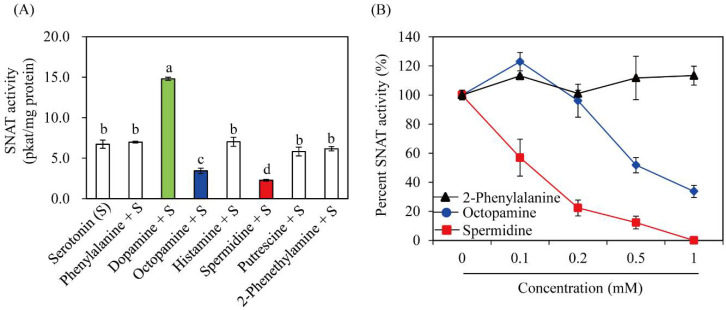
Effects of various substrates on SNAT activity. (**A**) SNAT enzyme activity of recombinant purified TvSNAT in the presence of serotonin (0.5 mM) and various amines (0.5 mM). Different letters indicate significant differences [*p* < 0.05; analysis of variance (ANOVA), followed by Tukey’s honest significant difference (HSD) post hoc tests]. The green, blue and red colors emphasize compounds which alter SNAT activity. (**B**) Dose-dependent inhibition of SNAT enzyme activity by spermidine or octopamine. SNAT activity was assayed in the presence of polyamines at various concentrations and expressed as a percentage relative to that in the absence of polyamines (Figure 4B). Data are the means ± standard deviation (*n* = 3).

SNAT enzymes can accept many other substrates, including phenylethylamines such as tyramine and indolethylamines such as tryptamine, serotonin, and 5-methoxytryptamine [35]. To determine whether TvSNAT can also acetylate other substrates, we measured SNAT enzyme activity in several other candidate substrates (Figure 3). The best substrates for the TvSNAT enzyme were tyramine (16.9 pkat/mg protein) and tryptamine (7.5 pkat/mg protein), followed by serotonin (6.7 pkat/mg protein) and 5-methoxytryptamine (0.7 pkat/mg protein) (Figure 3B). Substrate preference toward tyramine was also reported in sheep SNAT (20,413 pkat/mg protein) and rice SNAT2 (140 pkat/mg protein) [27]. TvSNAT exhibited the lowest enzyme activity toward 5-methoxytryptamine, whereas sheep and yeast SNAT showed the highest enzyme activity toward 5-methoxytryptamine [20], suggesting that melatonin biosynthesis via serotonin to 5-methoxytryptamine to melatonin is a less likely pathway in Archaea. In addition to these preferred arylalkylamines, other arylakylamines (dopamine, octopamine, 2-phenylethylamine, and histamine) and polyamines (spermidine and putrescine) were tested for possible acceptance as substrates for TvSNAT. Due to the lack of commercially available standard compounds of those acetylated substrates, we performed a SNAT inhibition assay (0.5 mM serotonin) in the presence of each substrate (0.5 mM) to determine whether SNAT activity is inhibited by co-incubation with one of these compounds. Spermidine strongly inhibited TvSNAT activity, followed by octopamine (Figure 4A). However, other compounds had no inhibitory effect on TvSNAT enzyme activity. These findings indirectly indicate that TvSNAT can acetylate spermidine and octopamine into *N*-acetylspermidine and *N*-acetyloctopamine, respectively. A dose-dependent inhibition assay showed that SNAT enzyme activity decreased to 50% in the presence of 0.1 mM spermidine; the same inhibition effect was observed at 0.5 mM octopamine (Figure 4B). Thus, TvSNAT appears to prefer spermidine to serotonin as a substrate. TvSNAT shares 9% sequence identity with human spermidine/spermine N^1^-acetyltransferase-1 [36], suggesting the bifunctional activity of TvSNAT toward serotonin and spermidine. This enhanced SNAT activity in the presence of dopamine was unexpected because recombinant fish SNAT enzyme activity was inhibited by co-incubation with dopamine [37]. The detailed enzymatic inhibition kinetics of TvSNAT require further study.

### 3.4. Phylogenetic Analysis

**Figure 5:**
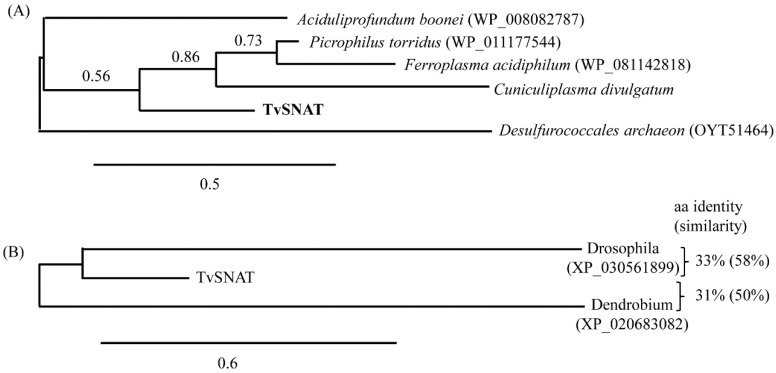
Phylogenetic analysis of TvSNAT constructed using the neighbor-joining method for (**A**) Archaea and (**B**) Animalia and Plantae. Scale bars in (**A**,**B**) represent 0.3 and 0.6 substitutions per site, respectively. Numbers in parentheses are GenBank accession numbers of corresponding genes. Alignments and the phylogenetic analyses were performed using the BLAST-Explorer tool (www.phylogeny.fr, accessed on 6 November 2019)).

A phylogenetic tree constructed on the basis of SNAT aa sequences from different organisms revealed that TvSNAT orthologs are widely distributed in many other Archaea families, including Picrophilaceae (*Picrophilus torridus*), Ferroplasmaceae (*Ferroplasma acidiphilum*), DHVE2 (*Aciduliprofundum boonei*), Cuniculiplasmataceae (*Cuniculiplasma divulgatum*), and Thermoprotei (*Desulfurococcales archaeon*) (Figure 5A). A non-redundant search of the National Center for Biotechnology Information and National Institutes of Health protein sequence databases (http://www.ncbi.nlm.nih.gov/, accessed on 24 April 2019) using the BLASTp program revealed that TvSNAT had the highest homology (33%) to *Drosophila novamexicana* (GenBank accession no. XP_030561899) among the kingdom Animalia, whereas among the kingdom Plantae, *Dendrobium catenatum* exhibited 31% identity to TvSNAT (Figure 5B). Further study is required to determine whether these animal and plant homologs of TvSNAT exhibit SNAT enzyme activity. 

### 3.5. Characterization of Transgenic Rice Plants

**Figure 6:**
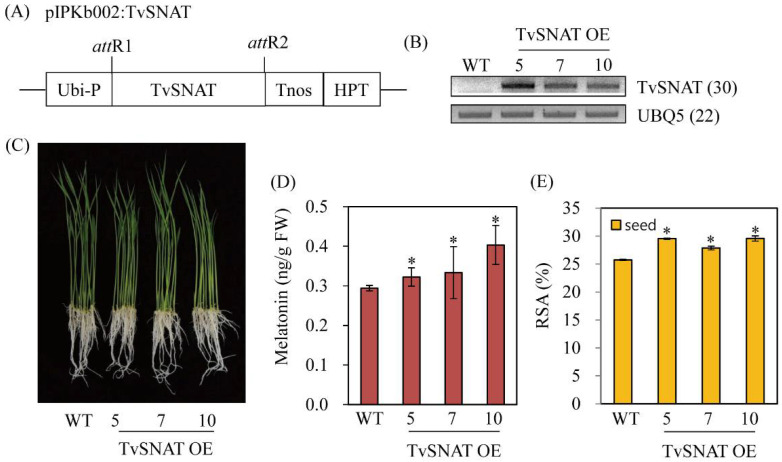
Schematic diagram of overexpression binary vectors, reverse-transcription polymerase chain reaction (RT-PCR) analyses, and melatonin content in transgenic rice. (**A**) Binary vector (GenBank accession no. EU161568) used for *TvSNAT* overexpression. (**B**) RT-PCR analyses of 7-day-old transgenic and wild-type (WT) rice seedlings. (**C**) Phenotypes of 7-day-old seedlings. (**D**) Melatonin content in 7-day-old seedlings. (**E**) Radical scavenging activity assessed by 1,1-diphenyl-2-picrylhydrazyl (DPPH) in seeds. *TvSNAT*, *Thermoplasma volcanium serotonin *N*-acetyltransferase*; *Ubi-P*, maize ubiquitin promoter; *HPT*, hygromycin phosphotransferase; *UBQ5*, rice ubiquitin 5 gene (GenBank accession no. Os03g13170). Numbers in parentheses indicate the numbers of PCR cycles performed. Asterisks indicate significant differences from the WT (Tukey’s honest significant difference test; *p* < 0.05). RSA, radical scavenging activity.

To gain further insight into the role of *TvSNAT* in melatonin biosynthesis in vivo, we generated transgenic rice plants overexpressing the *TvSNAT* gene under the control of the maize ubiquitin promoter (Figure 6A). Eleven independent T_1_ transgenic rice lines were initially screened on half-strength Murashige and Skoog medium containing 50 μg/mL hygromycin. Among these lines, one-copy transgene insertion lines with a 3:1 hygromycin segregation ratio were further selected and selfed to produce T_2_ seeds. Three homozygous transgenic rice plants overexpressing *TvSNAT* (TvSNAT-OE) were used for further analysis. Transgenic plants showed ectopic overexpression of transgenes in rice plants according to reverse-transcription (RT)-PCR analyses (Figure 6B). Three 7-day-old TvSNAT-OE transgenic rice seedlings produced more melatonin than wild-type (WT) seedlings (Figure 6C,D), indicating that *TvSNAT* gene overexpression was functionally coupled to enhanced melatonin production in rice plants. To see whether melatonin increase is closely coupled to increased antioxidant activity, the radical scavenging activities were investigated from the transgenic and wild-type seeds. As expected, the TvSNAT-OE lines exhibited high antioxidant activities to quench DPPH radicals compared to wild type (Figure 6E). On average, the transgenic lines showed 30% radical scavenging activity, whereas wild type had 25% radical scavenging activity, indicative of 12% higher antioxidant activity in the TvSNAT-OE lines. 

**Figure 7:**
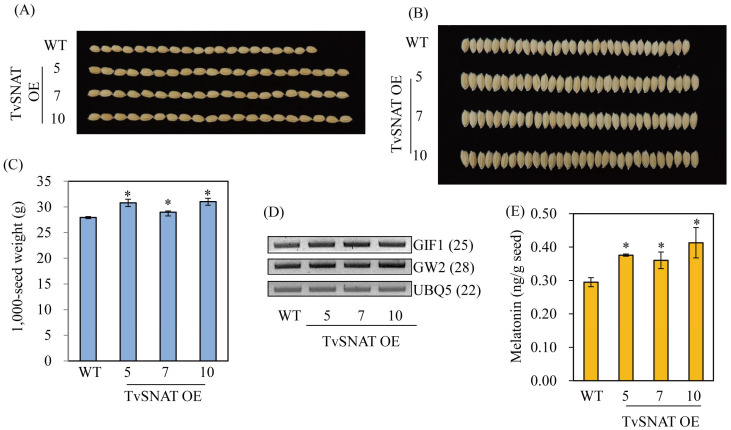
Rice grain morphology and expression levels of seed size-related genes. (**A**) Grain lengths, (**B**) grain widths, and (**C**) 1000-grain weights of the wild-type (WT) and TvSNAT-OE lines. (**D**) Expression levels of rice seed size-related genes determined by reverse-transcription polymerase chain reaction (RT-PCR) in 7-day-old seedlings. (**E**) Seed melatonin content levels. GenBank accession numbers for *GIF1*, *GW2*, and *UBQ5* are Os04g33740, Os02g14720, and Os03g13170, respectively. Numbers in parentheses indicate the numbers of PCR cycles performed. Asterisks indicate significant differences from the WT (Tukey’s honest significant difference test; *p* < 0.05).

Enhanced melatonin synthesis in the TvSNAT-OE transgenic rice plants led to increased grain size due to increased seed length and width compared with the WT control (Figure 7A,B). Similarly, 1000-seed weight was higher in transgenic seeds than in WT seeds (Figure 7C). We investigated two representative genes responsible for controlling rice seed size: *GRAIN INCOMPLETE FILLING 1* (*GIF1*) is a positive factor and *GRAIN WIDTH 2* (*GW2*) is a negative factor in controlling rice seed size. *GIF1* expression was higher in the TvSNAT-OE lines than in the WT, whereas *GW2* expression did not differ between the TvSNAT-OE and WT lines (Figure 7D). Melatonin levels were also increased in seeds of the TvSNAT-OE lines compared with the WT (Figure 7E). This is the first report of enhanced grain size in transgenic rice plants caused by an increase in endogenous melatonin, whereas small grain size was previously observed in various transgenic rice plants downregulating melatonin synthesis [38]. These data indicate that melatonin is positively associated with the control of grain size in plants. Further in-depth studies are required to investigate the roles of TvSNAT in rice growth, development, yield, and defense responses against environmental stresses.

## 4. Discussion

It has long been postulated that archaeans may be capable of synthesizing melatonin; however, no evidence to support this hypothesis has been discovered to date. Similarly, no enzymatic evidence has been discovered to confirm the existence of archaean melatonin biosynthetic genes such as *SNAT*, although archaeans contain approximately 200 genes belonging to the GNAT family [25]. Among these, two *GNAT* genes were suggested to be potentially capable of melatonin synthesis on the basis of sequence homology [2,25,26]; however, no direct evidence has been obtained through either enzymatic or mutant analysis.

In this study, we initially searched for *SNAT* genes encoding SNAT enzyme activity among archaean GNAT family members. SNAT is the penultimate enzyme of the melatonin biosynthesis pathway; it catalyzes serotonin into *N*-acetylserotonin, followed by melatonin synthesis through *O*-methyltransferase enzymes [14]. As SNAT plays a rate-limiting role in melatonin biosynthesis in both animals and plants [1], SNAT identification from archaeans could provide strong evidence for the presence of melatonin in archaea. Commensurate with the pivotal role of *SNAT* genes, it is highly likely that Gram-positive bacterial *SNAT* genes are ancestral to present animal *SNAT* genes, while cyanobacterial *SNAT* genes are ancestral to plant *SNAT* genes, suggesting an essential role of *SNAT* genes in melatonin biosynthesis among all living organisms [15,35]. 

In this study, we employed three archaean *SNAT* candidate genes from the GNAT family, including two recommended genes possessing NAT activity [25,26]. Using recombinant GNAT enzymes, we discovered a *SNAT* gene in *T. volcanium* that was previously annotated as a TvArd1 [26]. Ard1 encodes NAT, which transfers an acetyl group from acetyl-CoA to the N-terminal of various proteins [39,40]. Although TvArd1 (or TvSNAT) exhibits NAT activity at 25 °C [26], aa sequence identity between TvArd1 and human Ard1 was <14%, and human Ard1 (235 aa) was larger than TvArd1 (154 aa), suggesting that *TvArd1* may not be an ortholog gene of human *Ard1*. 

In common with TvArd1, *Arabidopsis thaliana* SNAT1 (AtSNAT1) exhibits acetyltransferase activity toward a series of substrates such as histone [41], chloroplast protein [42], and arylalkylamines [14,43]. Of note, AtSNAT1 exhibited SNAT activity within a broad range of temperatures (25–55 °C), as well as high serotonin affinity (*K*_m_ = 309 μM). The melatonin biosynthetic roles of AtSNAT1 were confirmed by gain- and loss-of-function analyses as well as exogenous melatonin treatment in view of pathogen defense [44], endoplasmic reticulum stress [5], high light stress [45], and flowering [23], suggesting that AtSNAT is more important to melatonin biosynthesis than to NAT or histone acetyltransferase.

*TvSNAT*-OE transgenic rice plants exhibited a large-seed phenotype, suggesting the involvement of melatonin in seed size regulation. Although larger seeds have not been observed in previous studies of transgenic rice plants producing higher melatonin levels than WT plants [46,47], other plants, including maize and cucumber, have shown increased seed or fruit size following exogenous melatonin treatment [48,49]. Intriguingly, transgenic rice plants overexpressing the rice *SNAT2* gene produce longer seeds than WT plants, although seed width remained unaffected [37]. These data suggest that melatonin is involved to some extent in rice seed size control. Several rice genes have been found to be responsible for controlling grain size [50,51] including *GRAIN SIZE 3* (*GS3*) [52], *GW2* [53], *GIF1* [54], and *RICE BIG GRAIN 1* (*RBG1*) [55]. GS3 and GW2 function as negative regulators of grain size, whereas *GIF1* and *RBG1* function as positive regulators. In this study, we found that melatonin-mediated larger grain size was mainly ascribed to the induction of *GIF1*, which encodes a cell wall invertase [54]. A previous study reported that melatonin treatment elevated cell wall invertase activity in *A. thaliana*, followed by pathogen resistance due to the increased cell wall reinforcement and callose deposition [56]. Further detailed mechanisms by which melatonin regulates seed size remain to be investigated in the near future. In addition to enhanced seed size, we anticipate that many other biological functions will be discovered through TvSNAT overexpression in rice plants, on the basis of the previous known roles of melatonin counteracting damages caused by biotic and abiotic stresses such as drought and salt [8,57,58,59]. 

## 5. Conclusions

This is the first study to discover the *SNAT* gene in Archaea. We demonstrated that the *TvSNAT* candidate gene from *Thermoplasma volcanium*, which was previously named *TvArd1*, exhibited SNAT enzyme activity. The highest amine substrate for TvSNAT was tyramine, followed by tryptamine, serotonin, and 5-methoxytryptamine, which were similar to those of plant SNAT enzymes. Homologs of *TvSNAT* have been found in many Archaea families. Ectopic overexpression of *TvSNAT* in rice was functionally coupled with the enhanced melatonin synthesis, resulting in the increased rice seed size. Consequently, one of key genes responsible for controlling rice seed size, *GIF1* was significantly overexpressed in the TvSNAT-OE lines compared to that in wild type. Our findings will open new avenues for research involving the cloning of *TvSNA*T orthologs in many different phyla, allowing the exploration of their functional roles and regulation of melatonin biosynthesis in living organisms. 

## Data Availability

The data presented in this study are available within the article.

## Supplementary Materials

The following are available online at https://www.mdpi.com/article/10.3390/antiox11030596/s1, Table S1: Sequences of primers used in this study. Table S2: Full length nucleotide sequences of three *GNAT* genes of both native and synthetic forms.
