# Supplementary material for: Functional Characterization of Serotonin N-Acetyltransferase in Archaeon Thermoplasma volcanium
Source: Antioxidants (Basel). 2022 Mar 21;11(3):596. doi: 10.3390/antiox11030596 (PMC8945778; doi:10.3390/antiox11030596)
Supplement: Supplementary file 1 [file antioxidants-11-00596-s001.zip › antioxidants-1618825-supplementary.pdf]

Supplemental Table S1. Sequences of primers used in this study

Gene	Forward (5´-3´)	Reverse (5´-3´)
Sequences of primers used for *E. coli* expression		
MaGNAT	AAA AAG CAG GCT CCA TGG TGA CCC TCG GCG	AGA AAG CTG GGT TCA GCA GGT GCG CTC CCC
PfGNAT	AAA AAG CAG GCT CCA TGA TCA TAG AGA AGG	AGA AAG CTG GGT TCA CGA GTT CTT GAA CTT
TvGNAT	AAA AAG CAG GCT CCA TGG CGA TCA ACG CGG	AGA AAG CTG GGT TCA CAC GAT CCG CCA CAT
attB	GGG GAC AAG TTT GTA CAA AAA AGC AGG CT	GGG GAC CAC TTT GTA CAA GAA AGC TGG GT
Sequences of primers used for transgenic rice plant		
TvSNAT	AAA AAG CAG GCT CCA TGG CGA TCA ACG CGG	AGA AAG CTG GGT TCA CAC GAT CCG CCA CAT
GIF1	CCC GCC GGC GAC GAG CAC CAC AT	CCG CCG GCC TGA ACA CCC TGA AGA
GW2	CAG CCA CCC AGT ATG GAC TT	GGT GAA CAA AAA GGC CAA GA
UBQ5	CCG ACT ACA ACA TCC AGA AGG AG	AAC AGG AGC CTA CGC CTA AGC
attB	GGG GAC AAG TTT GTA CAA AAA AGC AGG CT	GGG GAC CAC TTT GTA CAA GAA AGC TGG GT

Supplemental Table S2. Full length nucleotide sequences of three *GNAT* genes of both native and synthetic forms. The nucleotide sequences of synthetic GNAT genes were codon-optimized with reference to the rice SNAT2 codon. Modified nucleotides were shown in bold.

Gene	Native nucleotide sequence	Modified synthetic nucleotide sequence
Ma GNAT	atggtgaccttgggggcgacatcgatatcttcgcagtcagtgaatgggatgccgaggcgcttcttgcgctctaccgcgcgggggatggtggaaggaggagtgggacccatcccacatcccggtcctggtcaggtccaccttctcattcgtggtagccgtgcaccggcagagcgggcggacggtcgggatggggagggcaatatcagacggcgtatcggatgcgtacattcaggacctcgtcgtcctccccgaattcaggaaccggggggtcggaaaggcgctccttatgaggcttgttgatatctgcagggccaggggggatatcctggatcgcgctcgttgccgagccgggaacgtccgacttttaccgggagtcggggttcgtggtcatggaaggccatattcccatgcgttaccagggaggggagcgcacgtgctga	atggtgacc**c**t**c**gg**c**gg**g**gacatcga**c**atcttcgc**gg**t**gtcg**ga**g**tggga**c**gc**g**gaggcgct**c**ct**g**gcgct**g**taccgcgcggg**c**gg**c**tggtggaaggaggagtgggaccc**g**tcccacatcccggt**g**ctggt**gc**g**c**tc**g**ac**g**ttctc**c**ttcgtggt**c**gc**g**gtgcaccg**c**cag**tcg**gg**c**cggac**c**gt**g**gg**c**atgggg**c**g**c**gc**g**at**c**tc**g**gacggcgt**g**tc**c**ga**c**gc**c**tacat**c**caggacct**gg**tcgt**g**ctccc**gg**a**g**ttc**c**g**c**aaccgggg**c**gt**g**gg**c**aaggcgctcct**g**atg**c**ggct**c**gt**gg**a**c**atctg**tc**g**c**gc**gc**g**c**gggat**c**tc**g**tggatcgcgctcgt**gg**c**g**gagccggg**c**acgtc**gg**actt**c**taccg**c**gagtcggggttcgt**c**gt**g**atgga**g**ggcca**c**at**c**cc**g**atgcg**c**taccaggg**c**g**g**ggagcgcac**c**tgctga
Pf GNAT	atgataattgaaaaagttaatgaaccaatcaagctaaaagatgaccttctaaaattcgtctttggagtttatcaaagcacaaatggagcgtatcccgccctagagtgggcagagaaaaagcccgaacccaacgattttgagggctttaagaaaatctatgagccatttttagagtttaggctcacaaaagaatttgatgaactctacgttgctaaggacaataacaatattattggaacagtagcattggtatacaacctaaaaggaaaggaagtttggtgggtgccagaagaacttagggagaaaaatatcggactaatcgaattctttatggtggatgaaaagtacagaggaaagagaatcgggagcaaacttcttacctttgcaatcaagagattaaaggaattaggaaaggaaatatatatcgtgacttttccccacttacccgcataccaatattatctaaagaaggggtttcagaaggtcatggactacaaagagttcgtgattctaaaattcaagaattcatga	atgat**c**at**a**ga**g**aa**g**gt**g**aa**c**ga**g**cc**g**atcaagct**c**aa**g**ga**c**gacct**c**ct**g**aa**g**ttcgt**g**tt**c**gg**c**gt**g**ta**c**ca**gtcg**ac**c**aa**c**gg**c**gcgta**c**cc**g**gc**g**ct**g**gagtggc**g**gagaa**g**aagcccga**g**cc**g**aacga**c**tt**c**gagggctt**c**aagaa**g**atcta**c**gagcc**g**tt**cc**t**g**gagtt**cc**g**c**ct**g**ac**c**aa**g**ga**g**tt**c**ga**c**ga**g**ctctacgt**g**gc**g**aaggacaa**c**aacaatat**c**at**c**gg**c**ac**g**gt**g**gc**gc**t**c**gt**c**tacaacct**g**aa**g**gg**c**aagga**g**gt**g**tggtgggtgcc**c**ga**g**ga**g**ct**cc**g**c**gagaa**g**aa**c**atcgg**g**ct**c**at**a**ga**g**ttctt**c**atggtgga**c**ga**g**aagtac**c**g**c**gg**g**aag**c**g**g**at**t**gg**ctcg**aa**g**ct**c**ct**g**ac**g**tt**c**gc**g**atcaagag**gctc**aagga**gc**t**g**gg**c**aagga**g**at**c**ta**c**atcgt**c**ac**c**tt**c**cc**g**cac**c**t**c**cccgc**g**tacca**g**ta**c**ta**c**ct**c**aagaaggg**c**tt**c**cagaa**a**gt**g**atggactacaaagagttcgtgat**c**ct**g**aa**g**ttcaagaa**c**tc**g**tga
Tv Ard1 (Tv SNAT)	atggctattaatgctgttgccggaactatcagagagttctcaccgaaagatatcgagagtgtttataggatagcacagacttccttaactgagtattatacgcaagccctgatacttgatctccacagagaatggccggaatcatttatggtttacactgtagctggttctgttgtcggttttatagtaggatcgaagtattcgaggacggaggccaggatattactcttcgcagtcgatgagaggttcaggagaatgggtgtaggcagcgcattgatggatgcattccttagcctgtgcagagagcagaacatgctcagcgtaaggctggaagtcaggacagacaatgacgaagctataagattctacaaaaaaatatggattcgtcattactgctatgctaccaaactattatagcgattcatccaatgcgtatacaatgtggcgtatagtttaa	atggc**g**at**c**aa**c**gc**g**gt**g**gc**g**gg**c**ac**c**atc**c**g**c**gagttctc**g**cc**c**aa**g**ga**c**atcgag**tcg**gt**g**ta**cc**g**c**at**c**gc**g**cagac**c**tc**gc**t**g**ac**c**gagta**c**ta**c**ac**c**ca**g**gc**g**ctgat**c**ct**c**ga**c**ctccac**c**g**c**ga**g**tggcc**c**ga**g**tc**c**tt**c**atggt**g**tacac**c**gt**g**gc**c**gg**c**tc**g**gt**g**gtcgg**c**tt**c**at**c**gt**g**gg**g**tcgaagta**c**tcg**c**g**c**acggaggc**g**aggat**cc**t**g**ctcttcgc**g**gtcga**c**gag**c**ggttc**c**gg**c**g**c**atggg**c**gt**g**ggc**tcg**gc**gc**tgatgga**c**gc**g**ttcct**gtcc**ctgtg**tc**gg**g**agcagaacatgctc**tcg**gt**gc**g**c**ctgga**g**gt**gc**g**c**ac**c**gacaa**c**gacga**g**gc**g**at**cc**g**ct**tctacaa**g**aa**g**ta**c**gg**c**ttcgt**g**at**c**ac**g**gc**g**atgct**g**cc**g**aacta**c**ta**ctcg**ga**c**tc**c**tc**g**aa**c**gcgta**c**ac**g**atgtggcg**g**atcgt**g**tga
